# Science in a crisis: conducting clinical trials in an emergency

**DOI:** 10.1186/1745-6215-16-S2-I3

**Published:** 2015-11-16

**Authors:** P Horby

**Affiliations:** 1 Centre for Tropical Medicine and Global Health, University of Oxford, UK

## 

In the closing plenary lecture, Prof Peter Horby describes the challenges of conducting clinical trials in emergency settings.

